# Recurrent abdominal wall dermatofibrosarcoma protuberans in a child: a challenging reconstruction

**DOI:** 10.1186/s40064-015-1125-1

**Published:** 2015-07-08

**Authors:** Karthik C Vallam, Monica Bhagat, Vinay Shankhdhar, Sajid S Qureshi

**Affiliations:** Division of Pediatric Surgical Oncology, Department of Surgical Oncology, Tata Memorial Centre, Ernest Borges Road, Parel, Bombay, 400012 India; Department of Plastic and Reconstructive Surgery, Tata Memorial Centre, Bombay, India

**Keywords:** Dermatofibrosarcoma protuberans, Children, Abdominal wall, Surgery, Reconstruction, Anterolateral thigh flap

## Abstract

**Introduction:**

Dermatofibrosarcoma protuberans is an uncommon low-grade soft tissue sarcoma with a high potential for recurrence as it has irregular finger like extensions.

**Case description:**

We report a case of a large, recurrent dermatofibrosarcoma protuberans in a child involving the anterior abdominal wall, which posed a challenge for reconstruction. Peritoneum sparing full thickness resection of the anterior abdominal wall, meshplasty and a free anterolateral thigh flap was performed for reconstruction of the defect.

**Discussion and evaluation:**

Large composite defect, involving more than half of the anterior abdominal wall, necessitate a free flap reconstruction. Although these reconstructions are technically challenging in children, they are the only option available.

**Conclusion:**

Complete surgical excision is essential for DFSP of the abdominal wall, which may result in large challenging defects. Free flaps remain the only option in this scenario and hence it is essential to have expertise for microvascular flap reconstruction.

## Background

Dermatofibrosarcoma protuberans (DFSP) is an uncommon, low grade soft tissue sarcoma of fibroblast origin. Surgical excision with negative resection margins is crucial to prevent recurrences (Bichakjian et al. 2014). The resultant soft tissue and skin defect often require reconstruction. Anterior abdominal wall defects are technically challenging to reconstruct especially in children since large donor areas are not available due to the small frame of children.

We present our experience in managing a child with recurrent DFSP of the anterior abdominal wall.

## Case description

**Figure 1:**
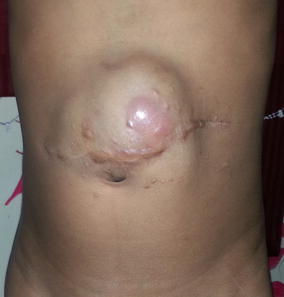
Clinical photograph.

**Figure 2:**
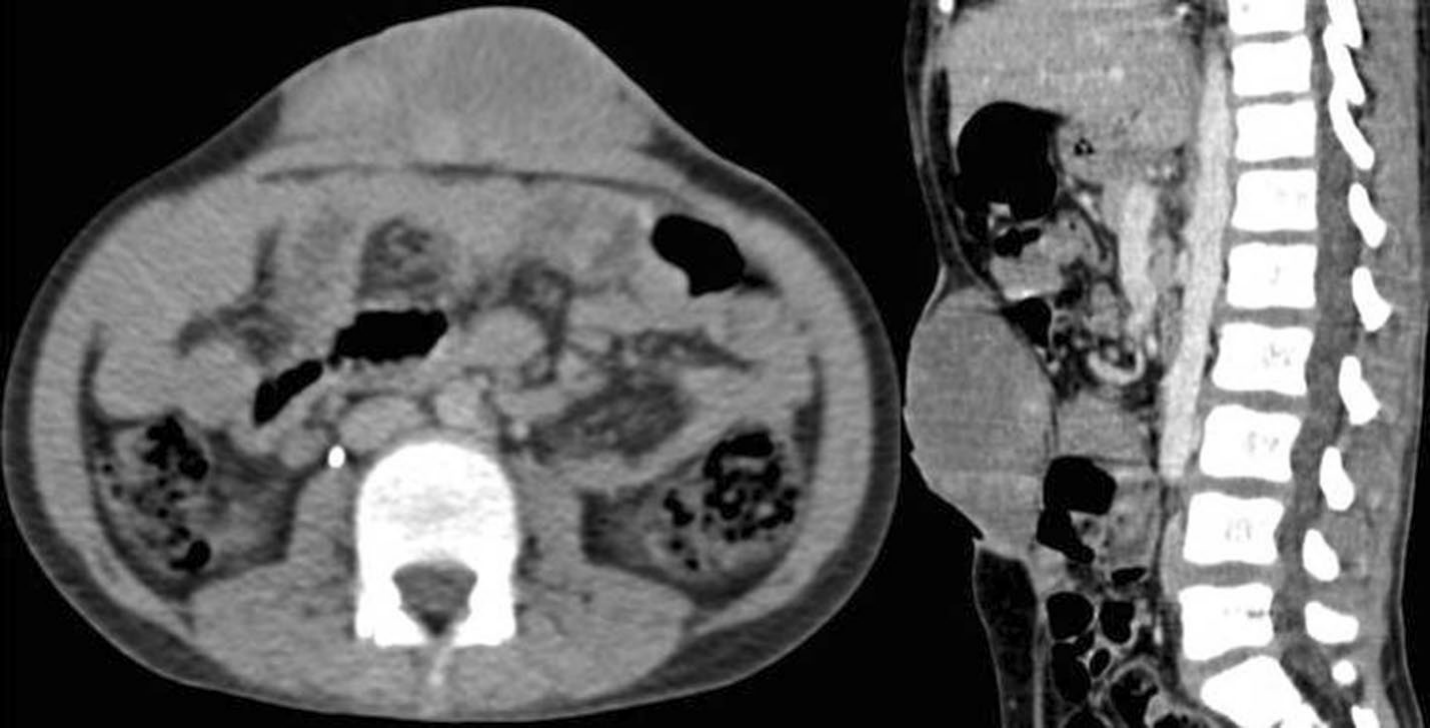
Computerized tomography images (axial and sagital) showing the lesion in the anterior abdominal wall.

A 10-year old boy presented with a recurrent abdominal wall tumor. The patient had an incomplete surgery 3 years earlier and had undergone two surgeries subsequently for recurrent swellings. There was neither history of trauma or any family history of similar swellings. Clinically the mass was 8 × 6 cm involving the skin and subcutaneous tissue with an 11 cm horizontal scar with prominent hatch marks and a separate drain site scar (Figure 1). Computerised tomography scan revealed a hypodense, ovoid mass measuring 3.8 × 7.9 × 8.4 cm infiltrating the rectus sheath with no obvious intra-abdominal extension (Figure 2).

**Figure 3:**
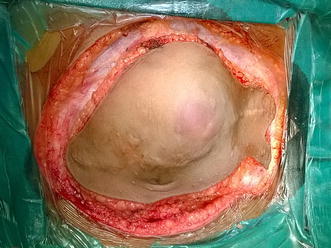
Surgical incision.

**Figure 4:**
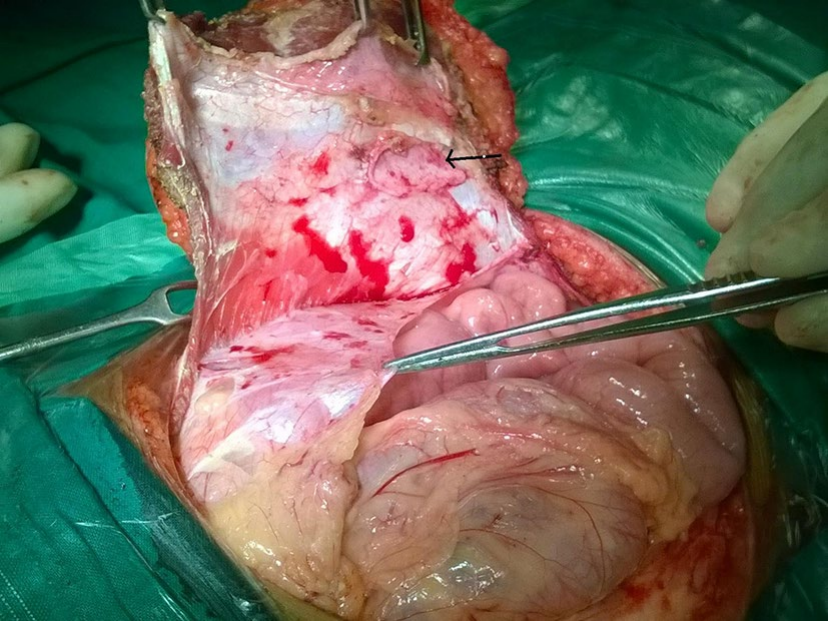
Full thickness abdominal wall resection preserving only the peritoneum. The *black arrow* shows the small strip of peritoneum which was excised with the specimen as there was suspicion of infiltration.

**Figure 5:**
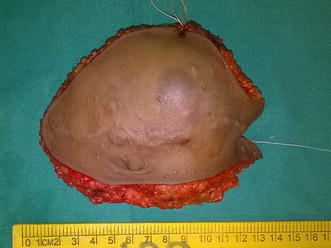
Resected specimen.

**Figure 6:**
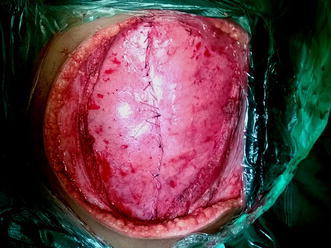
Primary closure of peritoneum.

**Figure 7:**
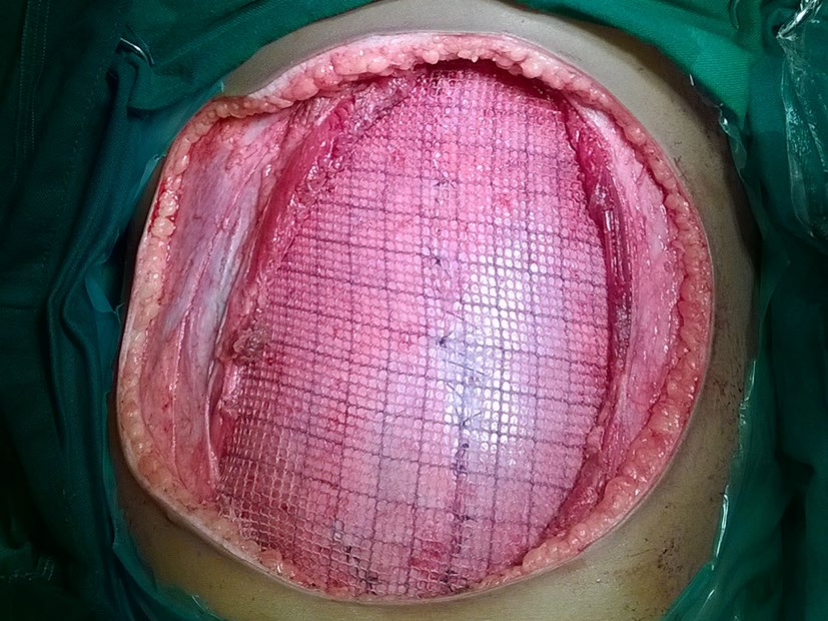
Pre-peritoneal mesh placement.

**Figure 8:**
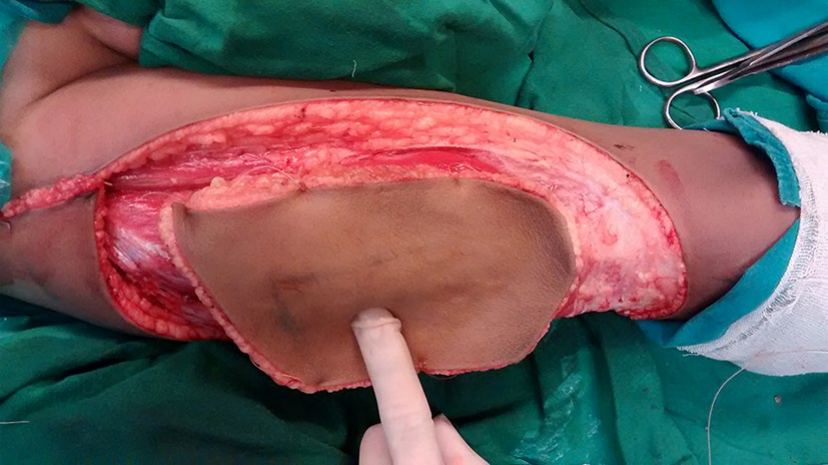
Harvested free anterolateral thigh flap.

**Figure 9:**
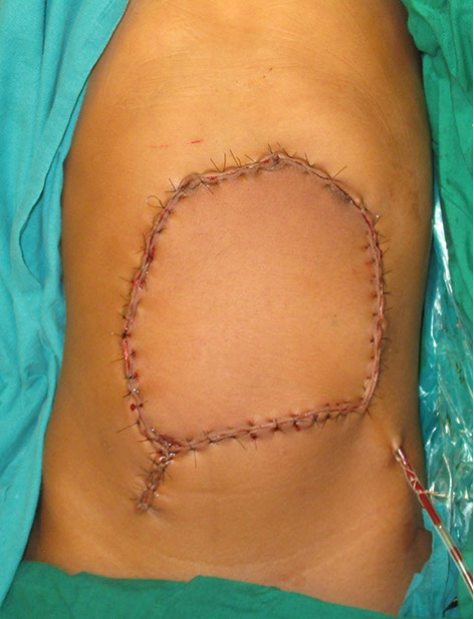
Immediate post-operative picture with the flap sutured to the abdominal wall defect.

Core needle biopsy was suggestive of DFSP. Surgical excision was planned with a wide margin of 2 cm and excising the entire rectus sheath (both anterior and posterior layers along with the muscles). The skin with the linear scar, hatch marks and the drain site scar were included in the resection (Figure 3). Since there was no intra-abdominal extension, the peritoneum was left intact except at one place where the tumor was abutting it (Figures 4, 5). Due to the sparse underlying omentum, placement of intraperitoneal mesh was deferred. The peritoneum was mobilized all around until the lateral abdominal wall and a primary closure of peritoneum was achieved (Figure 6). A pre-peritoneal VYPRO^®^ mesh was placed and anchored to the peritoneum (Figure 7). A free anterolateral thigh flap was harvested from the thigh and microvascular anastamosis was performed between the flap vasculature (cutaneous perforators of the descending branch of the lateral femoral circumflex vessels) and the deep inferior epigastric vessels (Figure 8). The deep fascia of the flap was sutured to the rectus sheath and skin approximated (Figure 9).

The patient had an uneventful recovery and was discharged from the hospital on the eighth postoperative day. All surgical resection margins were negative. The closest margin was 2 mm microscopically although grossly it was 12 mm away. The patient received postoperative radiotherapy (5,040 cGy/28#). At first follow up after three months the flap is well healed and the patient is disease free.

## Discussion

DFSP is a locally aggressive tumor with a high potential for recurrence as it has irregular finger like extensions (Bichakjian et al. 2014). The closest margin in our case was grossly 12 mm away from the tumour, however, on microscopic examination the tumor was extending till 2 mm from the cut margin indicating microscopic spread beyond grossly visualized disease which is difficult to assess intra-operatively. Due to this penchant of DFSP for microscopic extension beyond the gross confines and prior recurrences a wide excision with 2 cm margin was planned.

Kawaguchi et al. (2004) had suggested a thick fascial barrier (like the posterior layer of the rectus sheath) to be equivalent to a 3 cm cuff of soft tissue. Applying this concept, the entire posterior rectus sheath was excised along with the peritoneum only where the tumour had infiltrated focally. Preserving the peritoneum was crucial as the omentum was sparse and the bowel would have been exposed to the mesh. Intra-peritoneally placed meshes are prone to complications like extensive adhesions leading to chronic pain and increased chance of intestinal obstruction, sinus formation, infection, enterocolic fistula, etc. In addition, there is no conclusive evidence to suggest that the newer mesh like expanded polytetrafluoroethylene (ePTFE) or acellular dermal matrices are better than the basic polypropylene mesh (Ramakrishna and Lakshman 2013). As our patient had a large composite defect, involving more than half of the anterior abdominal wall, a free flap was the only feasible option. Free flaps that have been used for reconstruction of such defects are latissimus dorsi myocutaneous flap, the tensor fascia lata flap and the anterolateral thigh flap. The pros and cons of each of these flaps have been enlisted in Table 1 (Serafin 1995). Irrespective of the choice of flap, long term complications should be minimized as survival is very good in DFSP.

**Table 1:** Available options for reconstruction of large abdominal wall defects

	Pros	Cons
Latissimus dorsi flap	Constant vascular anatomy with long pedicle and large diameter	Significant motor deficit at donor site
	Donor defect can be closed primarily if skin paddle required is small—better cosmesis	Available skin paddle is small though muscle bulk is good
Anterolateral thigh flap	Long vascular pedicle with relatively large diameter	Donor site cosmesis is poor
	Large skin paddle	
Tensor fascia lata flap	Consistent, lengthy vascular pedicle	Donor site cosmesis is poor
	No significant functional loss at donor site	Bulky flap

## Conclusion

Complete surgical excision is essential for DFSP of the abdominal wall which may result in large challenging defects. Free flaps remain the only option in this scenario and hence it is essential to have expertise for microvascular flap reconstruction.
